# Phagocytosis at a glance

**DOI:** 10.1242/jcs.263833

**Published:** 2025-07-01

**Authors:** Manon Depierre, Chiara Pompili, Florence Niedergang

**Affiliations:** Université Paris Cité, CNRS, Inserm, Institut Cochin, F-75014 Paris, France

**Keywords:** Actin, Clearance, Internalisation, Degradation, Macrophages, Receptors

## Abstract

Phagocytosis functions as the internalisation mechanism responsible for engulfing large particles, microorganisms and cellular debris. It relies on specific cell surface receptors to induce membrane deformation, extension and contraction for particle engulfment. The actin cytoskeleton provides the necessary force for membrane deformation, whereas intracellular compartments aid in membrane reshaping and signal coordination. Following internalisation within a sealed compartment, the phagosome undergoes fusion and fission processes, ultimately forming a phagolysosome, where degradation takes place. Finally, a resolution step enables the recycling and reuse of soluble elements and membranes. This scavenging process is essential for feeding in single-celled eukaryotes and plays a crucial role in maintaining tissue homeostasis and regulating immune responses in higher eukaryotes.

## Teaser

**Summary:** Phagocytosis involves receptor-driven actin and membrane remodelling to engulf microbes and debris. The phagosome then matures for degradation, supporting both nutrition and immune responses.

**Figure:**
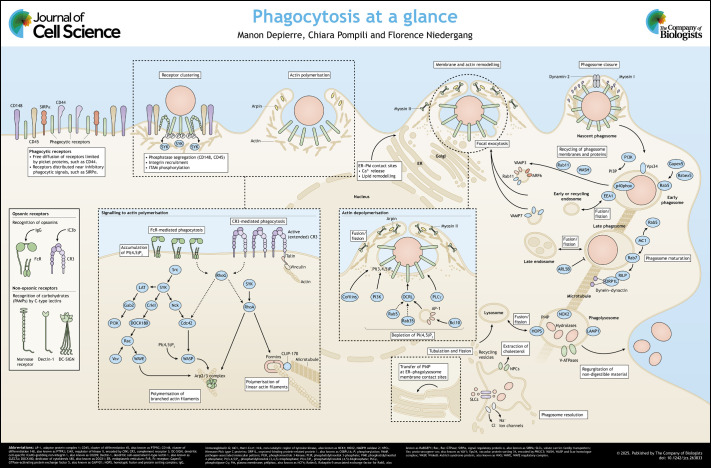
See supplementary information for a high-resolution version of the poster.

## Introduction

Phagocytosis is a universal cellular process that begins with receptor-mediated recognition and binding of particles larger than 0.5 µm, followed by their internalisation and degradation. Single-celled eukaryotes, such as the social amoeba *Dictyostelium discoideum* and other protozoa, rely on phagocytosis for feeding (Dunn et al., 2017; Guallar-Garrido and Soldati, 2024). In multicellular organisms, this process plays a crucial role in host defence against pathogens and is integral to immune and inflammatory responses.

In mammals, phagocytosis is a defining feature of specialised cells, including macrophages, dendritic cells and polymorphonuclear neutrophils. These cells, collectively known as professional phagocytes (see Glossary), engulf large particles, microorganisms and cellular debris, maintaining homeostasis. Beyond immune defence, phagocytosis is essential for tissue remodelling, normal cellular turnover, and the clearance of dead cells during development and adulthood (Doran et al., 2020; Morioka et al., 2019; Poon et al., 2014; Westman et al., 2019). For instance, retinal epithelial cells clear fragments shed by photoreceptor cells to maintain normal vision, whereas thyroid and bladder epithelial cells and kidney mesangial cells also exhibit phagocytic activity. In this Cell Science at a Glance article and the accompanying poster, we will focus on phagocytosis, excluding macropinocytosis (see Glossary), which, despite molecular similarities, operates independently of surface receptors (Canton, 2018; Salloum et al., 2023).

Glossary **Professional and non-professional phagocytic cells**

Some cells of the immune system, including dendritic cells, monocytes, macrophages and polymorphonuclear neutrophils, perform phagocytosis very efficiently. Certain non-immune cells, initially referred to as non-professional phagocytes, can also perform phagocytosis, either occasionally or routinely; for example, retinal epithelial cells efficiently phagocytose cell debris. The phagocytosis of cell debris has been called ‘efferocytosis’.

**Efferocytosis**

Efferocytosis (from *efferre*, Latin for ‘to take to the grave’, ‘to bury’) is the process of internalising dead cells or cellular debris. Apoptotic cells and debris express surface ligands that are recognised by specialised receptors on phagocytic cells.

**Macropinocytosis**

Macropinocytosis is a form of fluid-phase endocytosis that allows the internalisation of large particles. It relies on actin- and PI3K-dependent formation of membrane ruffles, which subsequently close to form intracellular macropinosomes. In immature dendritic cells, macropinocytosis occurs constitutively and is shut down when dendritic cells are mature, as opposed to other endocytosis pathways such as receptor-dependent phagocytosis. In other cell types, macropinocytosis can be induced by growth factors, viruses or bacteria.

**Opsonisation**

Opsonisation (from Greek, meaning ‘to prepare a meal’) is the process by which particles are coated with molecules called opsonins, making them capable of being bound and ingested by phagocytic cells. Common opsonins include host serum factors such as immunoglobulins and components of the complement cascade.

**Antigen presentation**

The presentation of antigens to stimulate T lymphocytes relies on two major classes of glycoproteins: MHC class I and class II. MHC class I proteins present peptides derived from the degradation of cytosolic proteins, whereas MHC class II proteins present peptides derived from the degradation of internalised exogenous material.

We describe the cell surface receptors that induce membrane deformation, extension and contraction for particle engulfment. The actin cytoskeleton provides the necessary force for membrane deformation, while intracellular compartments aid in membrane reshaping and signal coordination. Following internalisation, the phagosome undergoes fusion and fission processes, maturing into a degradative compartment called the phagolysosome. Finally, either undigested material is regurgitated, or a resolution step recycles degraded elements and membranes, reforms lysosomes and restores the readiness of the cell for subsequent phagocytic events.

## Phagocytic receptors

Phagocytosis is a receptor-mediated process that enables the uptake of various targets, from pathogens to apoptotic cells and debris. Professional phagocytes, such as macrophages, neutrophils and dendritic cells, are different types of immune cells that express a wide variety of membrane-bound receptors to recognise and engulf particulate matter (see Glossary).

Phagocytic receptors are broadly classified into two groups: opsonic receptors and non-opsonic receptors (see poster). Opsonic receptors engage targets coated with host-derived opsonins, such as antibodies and complement peptides (see Glossary), whereas non-opsonic receptors include pattern-recognition receptors (PRRs) and apoptotic cell receptors. PRRs directly bind pathogen-associated molecular patterns (PAMPs) on target surfaces (Kerrigan and Brown, 2009; Underhill and Ozinsky, 2002). Phagocytosis has been extensively studied using opsonic receptors as models, particularly Fcγ receptors (FcγRs), which recognise the Fc portion of immunoglobulin G (IgG), and complement receptor 3 (CR3; also known as CD11b/CD18, αMβ2 and Mac-1), which is a β2-family integrin that primarily binds the complement fragment iC3b. (Freeman and Grinstein, 2014).

Among the PRRs involved in microbial clearance, C-type lectin receptors (CLRs) recognise carbohydrates on fungal and bacterial walls through their C-type lectin-like domains (CTLDs). Two main members of the large CLR family are Dectin-1 (also known as CLEC7A) and the mannose receptor (MR, also known as CD206 and MRC1). Dectin-1 has a single CTLD that binds β-glucans, whereas the MR contains a cysteine-rich domain, a fibronectin type II domain and eight CTLDs that recognise mannose, fucose and *N*-acetylglucosamine (Kerrigan and Brown, 2009). The MR is important for the uptake of pathogens like *Mycobacterium tuberculosis* and *Pneumocystis,* but signalling pathways downstream of the MR that control phagocytosis remain unclear (Rajaram et al., 2017; Zhang et al., 2005). Moreover, the MR lacks a signalling motif in its cytoplasmic tail, suggesting that the ability of the MR to trigger phagocytosis depends on interactions with other signalling partners (Rajaram et al., 2017). Other C-type lectin receptors, such as Dectin-2 (also known as CLEC6A) and Mincle (also known as CLEC4E), signal through the association of the FcRγ chain (also known as FCER1G), a signalling adaptor primarily associated with Fc receptors, which contains immunoreceptor tyrosine-based activation motifs (ITAMs) (Sato et al., 2006; Yamasaki et al., 2008). Interestingly, a class C G-protein-coupled receptor, identified as the first PRR in these cells, has been shown to recognise folate and lipopolysaccharide and mediate the uptake of Gram-negative bacteria in the amoeba *D. discoideum* (Pan et al., 2018).

Phagocytes also play a crucial role in maintaining homeostasis by displaying a diverse array of receptors that mediate clearance of apoptotic cells in a process known as efferocytosis (see Glossary). This involves receptors that recognise the phospholipid phosphatidylserine (PS), which is a primary ‘eat-me’ signal on the membrane of dying cells. Although these mechanisms are crucial, this Cell Science at a Glance does not cover their signalling pathways, which might differ from those governing phagocytosis of pathogens (Boada-Romero et al., 2020; Mehrotra and Ravichandran, 2022; Moon et al., 2023).

## Receptor clustering

Despite the diversity of phagocytic receptors, some general principles govern the initiation of engulfment. Target recognition triggers receptor reorganisation into micro-clusters, a key event that promotes signalling for particle internalisation (see poster) (Li and Yu, 2021). The clustering of signalling molecules is an evolutionarily conserved phenomenon and is well described in the context of phagocytosis, particularly during FcγR-mediated uptake.

In resting macrophages, FcγRs are distributed in nanometre-scale clusters near the phagocytosis inhibitory receptor signal regulatory protein α (SIRPα, also known as SIRPA) (Lopes et al., 2017). Their free diffusion is physically constrained by transmembrane proteins like CD44, which acts as pickets in the cell membrane through tethering to the actin cytoskeleton (Freeman et al., 2018). Upon binding IgG-opsonised particles, FcγRs reorganise into micrometre-sized clusters, leading to phosphorylation of ITAMs in their cytoplasmic domains. This recruits and activates the spleen tyrosine kinase SYK, driving receptor displacement, actin remodelling and downstream signalling (see poster) (Jaumouille et al., 2014; Lin et al., 2016; Smith and Syme, 1982). Efficient engulfment initiation is influenced by lateral receptor mobility, ligand density and spatial distribution on the target surface. Recent studies show that macrophages sense ligand spacing, with tightly clustered ligands boosting receptor phosphorylation and improving phagocytic efficiency (Kern et al., 2021).

Concomitantly with receptor clustering, inhibitory molecules such as the tyrosine phosphatases CD45 (also known as PTPRC) and CD148 (also known as PTPRJ) must be excluded from the nascent phagocytic cup – a cup-shaped membrane indentation – to sustain receptor activation. Although these phosphatases can positively regulate downstream kinases, their long-lasting presence may otherwise impair receptor signalling (Goodridge et al., 2011; Zhu et al., 2008). First described for Dectin-1, this exclusion mechanism also applies to FcγRs. In the case of FcγRs, CD45 segregation is strongly dependent on integrin activation, which enhances receptor avidity for the target and forms a ‘diffusional barrier’ at the phagocytic site (Freeman et al., 2016).

## Signalling to actin polymerisation

Phagocytosis requires actin polymerisation, as it provides the necessary force to drive membrane deformation around the target. Engaged phagocytic receptors activate signalling pathways that recruit and activate actin filament nucleators, namely the actin-related protein 2/3 (Arp2/3) complex and formins (see poster) (Mylvaganam et al., 2021). These signalling pathways and the actin regulators involved can vary depending on the context and the target type, making phagocytosis a flexible process. The phagocytic machinery is best characterised downstream of FcγR and CR3 engagement; however, how other phagocytic receptors trigger actin polymerisation remains incompletely understood.

Small GTPases of the Rho family (Rho, Rac and Cdc42) regulate actin polymerisation during phagocytosis: classically, Rac and Cdc42 GTPases are thought to drive actin reorganisation during FcγR-mediated phagocytosis (Caron and Hall, 1998). CR3-mediated uptake was initially found to rely primarily on RhoA; however, more recent work suggests that CR3 can also activate signalling pathways similar to those triggered by FcγRs, depending on the context, as will be discussed in this section. RhoG plays a lesser understood role in both types of phagocytosis (Caron and Hall, 1998; Tzircotis et al., 2011).

In FcγR-mediated phagocytosis, receptor engagement and clustering of FcγRs triggers ITAM phosphorylation by Src-family kinases, creating docking sites for proteins with Src homology 2 (SH2) domains (Niedergang and Grinstein, 2018). The kinase SYK phosphorylates multiple substrates, creating additional docking sites for adaptor proteins such as linker for activation of T cells (LAT), non-catalytic region of tyrosine kinase (Nck, also known as NCK1) and regulator of kinase II (CrkII, encoded by *CRK*) (Gu et al., 2003; Kiefer et al., 1998). Recruitment and phosphorylation of LAT recruits the adaptor protein GRB2-associated binding protein 2 (Gab2), which in turn engages the p85 subunit of type I phosphoinositide 3-kinase (PI3K; p85 subunit encoded by *PIK3R*1) (Moon et al., 2005), leading to the local accumulation of phosphatidylinositol (3,4,5)-trisphosphate [PI(3,4,5)P_3_] and consumption of phosphatidylinositol (4,5)-bisphosphate [PI(4,5)P_2_]. In parallel, Nck promotes Cdc42 and Wiskott–Aldrich syndrome protein (WASP, also known as WAS) recruitment, while CrkII recruits dedicator of cytokinesis 180 (DOCK180, also known as DOCK1), which activates Rac GTPases (Lee et al., 2007). The guanine-nucleotide-exchange factor (GEF) Vav (also known as VAV1) also activates Rac GTPases downstream of FcγR engagement (Patel et al., 2002). WASP is activated by binding to GTP-bound Cdc42 and PI(4,5)P_2_ (Dart et al., 2012), a phosphoinositide generated early after receptor engagement (Botelho et al., 2000). In turn, WASP activates the Arp2/3 complex, which mediates the polymerisation of branched F-actin (May et al., 2000; Rotty et al., 2017). Interestingly, a more recent study has reported that FcγR-mediated phagocytosis is slower but is not impaired in *Arpc2^−/−^* macrophages, despite complete loss of Arp2/3 function (Rotty et al., 2017). This suggests that Arp2/3 is not strictly required for FcγR-mediated phagocytosis and that other actin regulators may compensate for its absence in this context.

CR3-mediated phagocytosis, by contrast, follows a distinct signalling pathway involving activation of RhoA and of the formin mDia1 (also known as DIAPH1), which interacts with the microtubule-associated cytoplasmic linker protein 170 (CLIP-170, also known as CLIP1) to facilitate phagocytic cup formation (Caron and Hall, 1998; Colucci-Guyon et al., 2005; Lewkowicz et al., 2008). CR3 engagement also leads to the assembly of signalling platforms at the phagocytic cup, which are enriched with adaptor proteins and kinases classically found in integrin-mediated focal complexes, such as talins, vinculin and SYK. In addition, SYK activity is required for CR3-mediated phagocytosis, and CR3 also depends on Arp2/3-mediated actin polymerisation (Jaumouille et al., 2019; May et al., 2000; Rotty et al., 2017; Walbaum et al., 2021). These findings suggest that CR3 can trigger different modalities of phagocytosis depending on the cellular context.

The signalling events triggered by other phagocytic receptors remain mostly unknown. The cytosolic region of the C-type lectin Dectin-1 contains a hemi-ITAM, suggesting that SYK could theoretically be recruited by two adjacent Dectin-1 molecules after phosphorylation by tyrosine kinases. However, the role of SYK in Dectin-1-mediated phagocytosis remains controversial (Kerrigan and Brown, 2009). Finally, receptors for apoptotic bodies vary considerably in how they trigger intracellular signalling. For example, the PS receptor brain-specific angiogenesis inhibitor 1 (BAI1, also known as ADGRB1) triggers the recruitment of the CrkII–engulfment and migration (ELMO)–DOCK180 module to activate Rac1, whereas T-cell immunoglobulin and mucin domain-containing protein 4 (TIM4) – a PS receptor – signals via integrins or other partners. These signalling pathways are conserved across species, as exemplified by the identification of some molecular players in *Caenorhabditis elegans* and *Drosophila* (Mehrotra and Ravichandran, 2022).

## Membrane remodelling

Efficient engulfment of large particulate material requires vesicular trafficking and targeted membrane delivery to the site of phagosome formation, a process termed ‘focal exocytosis’ (see poster), which also takes place during neurite outgrowth. The concept of focal exocytosis stems from the observation that plasma membrane surface area increases upon phagocytosis instead of decreasing (Hackam et al., 1998; Holevinsky and Nelson, 1998). Evidence that focal exocytosis is necessary for optimal phagocytosis has been provided by studies interfering with fusion machineries composed of vesicle soluble *N*-ethylmaleimide factor attachment protein receptors (v-SNAREs), a family of small, conserved eukaryotic proteins that mediate membrane fusion between organelles and the plasma membrane (Jahn et al., 2024). Many intracellular compartments have been shown to contribute to focal exocytosis, including recycling endosomes bearing vesicle-associated membrane protein (VAMP) 3 on their surface (Bajno et al., 2000; Braun et al., 2007; Niedergang et al., 2003), late endocytic VAMP7^­^-positive compartments and lysosomes (Braun et al., 2004; Czibener et al., 2006), and membranes derived from the Golgi and endoplasmic reticulum (ER) (Aderem, 2002; D'Amico et al., 2021; Gagnon et al., 2002), with ERS24 (also known as SEC22B) acting as a SNARE on the ER (Becker et al., 2005). The ER also contributes via membrane contact sites, facilitating local Ca^2+^ release and signalling, as well as lipid remodelling (Ghavami and Fairn, 2022; Nunes-Hasler and Demaurex, 2017). Small GTPases from the ARF and Rab families regulate the recruitment and fusion events of the compartments at the plasma membrane, with Rab11 proteins and ARF6 controlling recycling endosome delivery (Cox et al., 2000; Damiani et al., 2004; Niedergang et al., 2003; Patel and Harrison, 2008). Therefore, the process of recruitment and fusion of intracellular compartments not only supplies membrane for phagosome formation, but also delivers specific signalling molecules, remodels the lipid composition of the plasma membrane and regulates actin dynamics.

## Cooperation between membrane and cytoskeleton, and actin remodelling

Precise coordination between actin polymerisation and membrane delivery is essential for phagosome formation. Actin remodelling and depolymerisation facilitate vesicle delivery through the cortical actin meshwork of the phagocytic cup (see poster). Actin-depolymerising factor (ADF)/cofilin-family proteins, which sever and depolymerise actin upon dephosphorylation, play a role in phagocytosis (Adachi et al., 2002). Similarly, Arp2/3 complex inhibitor (Arpin), a negative regulator of the Arp2/3 complex, ensures efficient phagocytic uptake by coordinating actin polymerisation – as it is located together with the Arp2/3 complex and branched actin – at extending membrane folds (Jubrail et al., 2020). Actin dynamics are tightly linked to phosphoinositide metabolism, particularly the depletion of PI(4,5)P_2_, which is a crucial step in phagocytosis (Niedergang and Grinstein, 2018). PI(4,5)P_2_ depletion can occur in the following ways: (1) a cessation in synthesis due to the detachment of phosphatidylinositol phosphate kinases from the phagosome; (2) hydrolysis by phospholipase C (PLC)γ, generating diacylglycerol and inositol (1,4,5)-trisphosphate; and (3) phosphorylation and conversion of PI(4,5)P_2_ into PI(3,4,5)P_3_ by class I PI3Ks, which are crucial for efficient phagocytosis of large particles (Cox et al., 1999). Additionally, the PI(4,5)P_2_ and PI(3,4,5)P_3_ phosphatase oculocerebrorenal syndrome of Lowe (OCRL) further hydrolyses PI(4,5)P_2_ and contributes to F-actin removal during phagocytosis, a mechanism that is conserved from mammalian cells to *D. discoideum* (Marion et al., 2012; Loovers et al., 2007). OCRL is recruited to phagocytic sites via adaptor protein complex 1 (AP-1)-positive compartments, under the regulation of the nuclear factor kappa B (NF-κB) signalling protein B-cell lymphoma/leukemia 10 (Bcl10) (Marion et al., 2012; Deschamps et al., 2013). Rab5 and Rab35 GTPases might also play a role in OCRL recruitment to limit F-actin accumulation (Egami et al., 2011). This intricate interplay between intracellular compartments and signalling pathways govern actin dynamics and turnover, ensuring efficient membrane extension and phagosome closure.

## Phagosome closure

The final step of internalisation involves the fusion of membrane extensions to enclose the phagocytic target. Although the precise mechanisms of membrane fusion and fission remain incompletely understood, actin filaments, membrane tension and crucial motor proteins – myosins and dynamin-2 – are major players (see poster) (Barger et al., 2019; Marie-Anais et al., 2016). More precisely, myosin IE (MYO1E) and myosin IF (MYO1F) localise to the tips of the phagocytic cup – a localisation also observed for MyoK in *D. discoideum* – linking the actin cytoskeleton to the plasma membrane (Barger et al., 2019; Dieckmann et al., 2010). This likely increases membrane tension, creates adhesion sites on the target and facilitates uptake. Furthermore, despite controversial observations, myosin II could be required for its contractile activity to promote phagocytic cup closure. Indeed, myosin II is crucial in the final stage of the process, when the phagocytic target has reached 90% of internalisation (Vorselen et al., 2021). Notably, these mechanisms are highly conserved in *D. discoideum*, where myosin I accumulates specifically at the site of phagocytic cup constriction, and where myosin II, dynamin A and actin dynamics cooperatively contribute to facilitate cup closure (Dieckmann et al., 2010). Ultimately, dynamin-2 drives membrane constriction and mediates the final membrane scission, ensuring efficient phagosome completion (Marie-Anais et al., 2016).

## Phagosome maturation

Once closed, the phagosome undergoes a series of fusion and fission events with compartments of the endocytic pathway in a process called maturation, ultimately leading to the degradation of internalised material (see poster) (Levin et al., 2016). Proteomics approaches have greatly contributed to our understanding of phagocytosis by providing a record of phagosomal protein composition over time (Desjardins et al., 1994; Gotthardt et al., 2006). In addition, comparison of the phagosome proteomes from distantly related species has revealed an evolutionarily conserved core machinery: ∼75% of the mouse phagosome proteome consists of proteins that have orthologues found in amoebae, whereas phagosome proteins with immune functions are associated with mammals (Boulais et al., 2010). Concurrently, phagosomes are transported from the cell periphery to the perinuclear region along microtubules, a process that is essential for proper maturation (Blocker et al., 1997).

Nascent phagosomes become early phagosomes by acquiring the small GTPase Rab5, which is activated by the GEFs Gapex5 and Rabex5 (also known as GAPVD1 and RABGEF1, respectively) (Kitano et al., 2008; Vieira et al., 2003). Rab5 effectors include the class III PI3K vacuolar protein sorting 34 (Vps34, encoded by *PIK3C3*), which produces phosphatidylinositol 3-phosphate (PI3P) at the phagosomal membrane (Kinchen et al., 2008; Vieira et al., 2001). PI3P recruits p40phox (also known as NCF4), which is a subunit of the NADPH oxidase NOX2 (Anderson et al., 2008), and early endosome antigen 1 (EEA1), which mediates tethering and fusion of Rab5-positive phagosomes with early endosomes (Christoforidis et al., 1999). The Mon1–Ccz1 (MC1) complex, which has been identified as the GEF for Rab7 in the endocytic pathway, facilitates Rab5 dissociation and Rab7 recruitment (Kinchen and Ravichandran, 2010), enabling fusion with late endosomes and the formation of late phagosomes. The Rab7 effector RILP, in association with ORP1L (also known as OSBPL1A), links phagosomes to the dynein–dynactin complex, facilitating microtubule-based transport (Harrison et al., 2003; Johansson et al., 2007). Once phagosomes acquire Rab7, production of phosphatidylinositol 4-phosphate (PI4P) by phosphatidylinositol 4-kinase 2A (PI4K2A) and association of the HOPS complex promote fusion with lysosomes, generating phagolysosomes (Jeschke and Haas, 2018; Lobingier and Merz, 2012). In addition, the ADP ribosylation factor-like GTPase 5B (ARL5B), which controls trafficking between endosomes and the trans-Golgi network, is required for efficient phagosome maturation and bacterial clearance in human macrophages (Faure-Dupuy et al., 2024).

Parallel to maturation, phagosome membrane and proteins are recycled through vesicle budding and fission (see poster). Rab11 and its effectors mediate early phagosome recycling to the plasma membrane (Damiani et al., 2004; Leiva et al., 2006). In addition, the retromer complex and the WASH complex, as well as dynamin, facilitate the recycling of plasma membrane proteins from phagosomes at different maturation stages (Buckley et al., 2016; Gopaldass et al., 2012).

Phagosomes progressively acquire degradative properties during maturation. They become acidic through the acquisition of proton pumping V-ATPases (Kissing et al., 2018), and phagolysosomes become enriched in hydrolases and lysosome-associated proteins (LAMPs) (Binker et al., 2007). The production of reactive oxygen species (ROS), thanks to the assembly and activation of the NADPH oxidase NOX2 (Bode et al., 2023), as well as the presence of other bactericidal compounds, are crucial features of phagolysosomes (Guallar-Garrido and Soldati, 2024). These mechanisms collectively contribute to cargo degradation and pathogen elimination (Dupre-Crochet et al., 2013; Peri and Nusslein-Volhard, 2008). However, the balance between acidification and ROS production varies among phagocytic cells and their phenotypes (Canton, 2014). In dendritic cells, NOX2 activity regulates phagosome acidification to promote antigen presentation (see Glossary), probably by preventing fast degradation of internalised material (Mantegazza et al., 2008). Some receptors, like C-type lectin domain family 9 member A (CLEC9A), signal to promote phagosomal rupture, allowing the degraded material to reach the cytosol and the major histocompatibility (MHC) class I antigen processing pathway (see Glossary) (Canton et al., 2021). Engagement of Toll-like receptors (TLRs) by the target further enhances presentation of phagocytosed antigens by dendritic cells (Alloatti et al., 2015; Blander and Medzhitov, 2006), but the mechanisms by which TLR signalling modulates phagosome maturation remain unclear.

## Phagosome resolution and regurgitation of non-digestible material

Considerable progress has been made in recent years to understand how phagosomes resolve (see poster). A recent study has shown that macrophages recycle metabolic intermediates more efficiently when phagocytosing dead rather than viable bacteria, providing amino acids for protein synthesis (Lesbats et al., 2025). Solutes resulting from the degradation of cargo are exported into the cytoplasm through solute carrier (SLC)-family transporters such as the SLC7 family of amino acid transporters (Mylvaganam and Freeman, 2023). Concomitantly, resolving phagosomes are fragmented through fission, generating vesicles that replenish the pool of lysosomes (Lancaster et al., 2021). Osmotically driven shrinkage, mediated by endomembrane ion channels, initiates the deformation of the phagosome membrane (Chadwick et al., 2024; Freeman and Grinstein, 2018). The extraction of cholesterol from ingested membranous targets by Niemann–Pick type C proteins (a process impaired in the lipid storage disorder Niemann–Pick disease) seems to be a rate-limiting step in phagosome resolution (Barreda et al., 2024). Membrane contact sites with the ER facilitate resolution by transferring PI4P from phagolysosomes to the ER, which in turn recruits kinesin-interacting proteins ARL8B and SKIP (also known as PLEKHM2) to drive membrane pulling and fission (Levin-Konigsberg et al., 2019). When macrophages engulf non-digestible materials, they can expel them through regurgitation (Di et al., 2002), although such materials can also persist within phagosomes in the long term (Lancaster et al., 2021). This non-lytic exocytosis pathway is called extrusion, exclusion or vomocytosis, as initially coined to describe the regurgitation of the fungus *Cryptococcus neoformans* (Chayakulkeeree et al., 2011). Additionally, the release of partially digested material through fusion of the phagolysosome with the plasma membrane, termed eructophagy, is enhanced by TLR4 signalling (Greene et al., 2022). Of note, exocytosis of undigested remnants is constitutive in amoebae, and this mechanism is conserved between *D. discoideum* and animal cells (Gilbert et al., 2017; Watkins et al., 2018).

## Conclusion and perspectives

Phagocytosis is a specialised uptake process that shares many features with the endocytic pathway, including the progression from early endosomes to late endosomes and lysosomes. However, it is distinguished by the ingestion of large particles destined for degradation and the early production of ROS within the closed compartment. Furthermore, the outcome of phagocytosis depends on the specific receptor activated, influencing intracellular signalling, cell activation and the ultimate fate of the ingested material. Although we have not covered LC3-associated phagocytosis in this Cell Science at a Glance article, the involvement of the autophagy machinery might be of importance in the regulation of debris clearance, cell activation and inflammation (Pena-Martinez et al., 2022). In addition, several highly conserved mechanisms of cell debris engulfment, which play crucial roles in tissue remodelling and neuron pruning during development, as well as in some pathologies and inflammatory contexts, are not discussed here.

Pathogens have evolved strategies to manipulate host signalling and trafficking, altering phagocytic and killing activities. While the diversity of these manipulations is vast and beyond the scope of this discussion, understanding these mechanisms is the focus of extensive research. Insights into how these pathways are hijacked could lead to novel strategies for restoring effective phagocytosis, enhancing pathogen clearance, and preventing infections or chronic inflammation.

## Poster

Poster
