# Supplementary figures and images for: Phagocytosis at a glance
Source: J Cell Sci. 2025 Jul 1;138(12):jcs263833. doi: 10.1242/jcs.263833 (PMC12273632; doi:10.1242/jcs.263833)

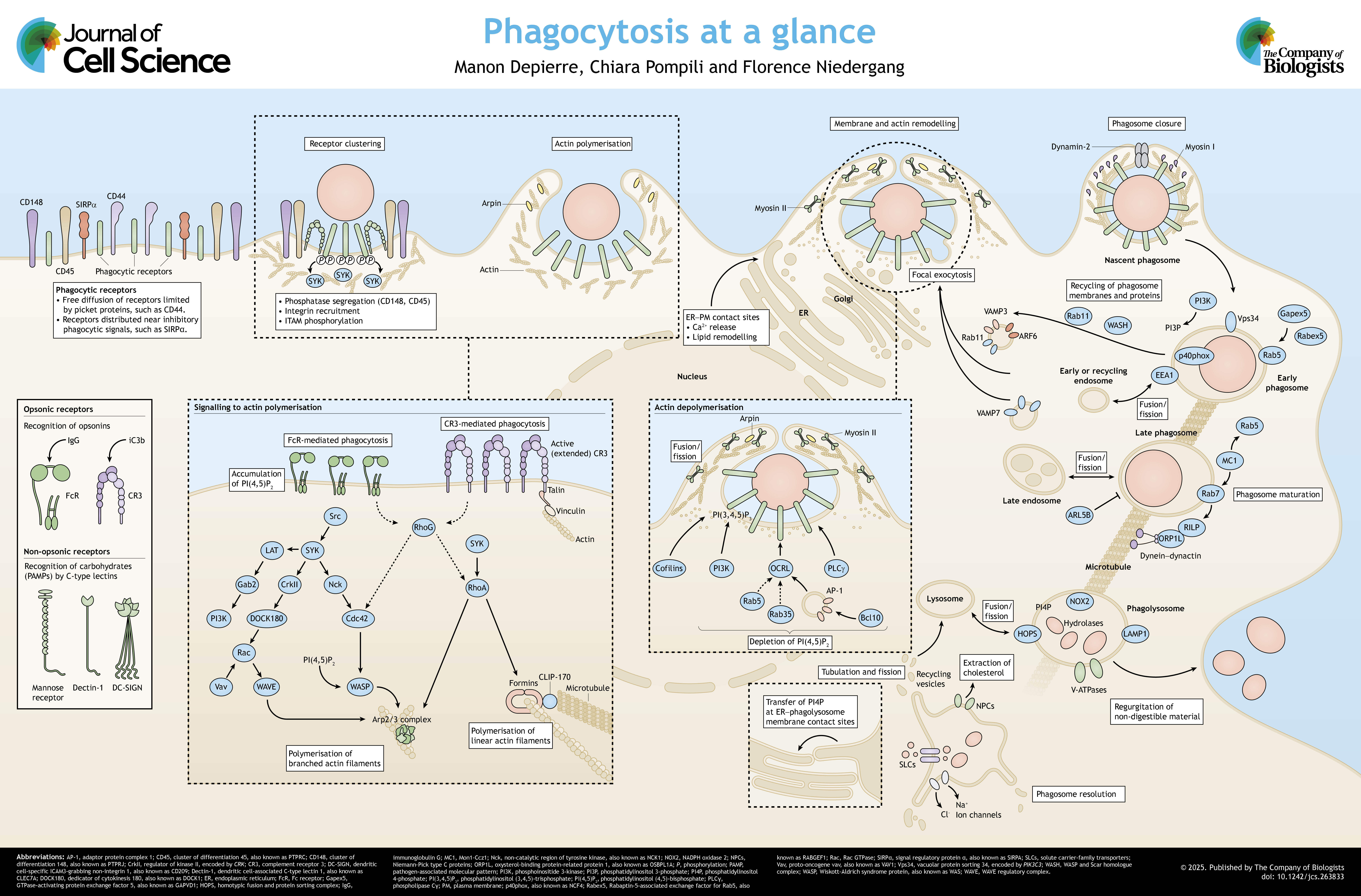

Supplement: Poster [file joces-138-263833-s1.jpg]
